# Toward an Improved Meningococcal Serogroup B Assay

**DOI:** 10.1128/mBio.00713-18

**Published:** 2018-05-15

**Authors:** Jan Poolman

**Affiliations:** a Janssen Vaccines & Prevention B.V., Bacterial Vaccine Discovery and Early Development, Leiden, Netherlands

**Keywords:** meningococcus serogroup B, *Neisseria meningitidis*, factor H binding protein, vaccines

## Abstract

Because of diverse sequences and differential expression of surface structures on individual invasive *Neisseria meningitidis* serogroup B (MenB) strains, predicting the efficacy of MenB vaccines using traditional human serum bactericidal assays (hSBA) is impractical. The meningococcal antigen surface expression (MEASURE) assay uses flow cytometry to quantitate the expression of factor H binding proteins (fHbp) contained in the bivalent rLP2086 MenB vaccine. To date, experience with MEASURE has been lacking, and in a long-awaited article, McNeil et al. (mBio 9:e00036-18, https://doi.org/10.1128/mBio.00036-18), provide detailed mapping of a cross-reactive antibody binding epitope and explore the potential utility of MEASURE in predicting the susceptibility of individual MenB strains to antibody-mediated killing. Remaining questions center around why some strains with high fHbp expression are nonsusceptible to anti-fHbp antibody killing. Consideration of alternative methods, such as a standardized enzyme-linked immunosorbent assay (ELISA), might offer a more readily available and reproducible assay for wider use.

## COMMENTARY

The first highly protective pediatric meningococcal vaccines (serogroup C protein conjugate vaccines) were licensed for use in 1999, followed rapidly by the development of multivalent meningococcal conjugate vaccines targeting up to four serogroups (A, C, W, and Y). It took another 14 years before the first broadly protective vaccines targeting serogroup B (MenB) became available. The development of MenB vaccines required a novel approach because the MenB polysaccharide turned out to be poorly immunogenic, probably because of similarities between the MenB capsular polysaccharide and a human poly-sialic acid present in fetal neural adhesion molecules. The two licensed MenB vaccines contain surface proteins capable of inducing bactericidal antibodies; rLP2086 (Trumenba: Pfizer) contains two factor H binding proteins (fHbp) (subfamily A and subfamily B), and 4CMenB (Bexsero; GlaxoSmithKline) contains one fHbp, neisserial heparin binding antigen, and *Neisseria* adhesin A, with an outer membrane vesicle vaccine from the NZ 98/254 strain (B:4:P1.7-2,4; sequence type 42 [ST-42] [cc41/44]). The sequences and expression levels of surface proteins on individual MenB strains are highly diverse, and the two vaccines faced similar challenges during evaluations of immunogenicity and predictions of efficacy during development. Because hundreds or potentially thousands of unique MenB strains exist, it is not feasible to test them individually by the hSBA using human complement. So while the percentage of individuals who reached a threshold hSBA antibody level was used to support licensure of both vaccines, other methods were needed to estimate vaccine coverage across multiple MenB strains. The manufacturers of both licensed MenB vaccines have developed assay systems with readouts derived from the expression level of each vaccine protein and its cross-reactivity with serum antibodies. For 4CMenB, the assay is called the meningococcal antigen typing system (MATS), which combines PorA genotyping with a sandwich ELISA to generate the “relative potency” against the vaccine surface proteins for individual tested strains. For rLP2086, the assay is called the MEASURE assay, which uses flow cytometry to quantitate the mean fluorescence intensity (MFI) indicating fHbp surface expression on individual MenB strains.

In a recent article in *mBio*, McNeil et al. (1) conducted a detailed analysis of the surface expression of meningococcal factor H binding proteins (fHbp) as measured using a nonbactericidal cross-reactive monoclonal antibody. The antibody epitope has been elegantly mapped in detail, and their paper adds significantly to our understanding of the immunobiology of fHbp. McNeil et al. then went on to validate the meningococcal antigen surface expression (MEASURE) assay and investigate fHbp expression on large collections of clinical MenB isolates, correlating fHbp expression with human serum bactericidal assays (hSBA) using pooled postvaccination serum. Interestingly, the correlation between bactericidal antibody titers and the level of fHbp detected in the MEASURE assay was poor. Nevertheless, the authors were able to show that bacteria were usually susceptible to bactericidal killing when the mean fluorescence intensity (MFI) was at least 1,000, which equated with 30 pg (dry weight) of fHbp per microgram of total cell protein. From a collection of 1,814 isolates, 91% were shown to express fHbp at this level.

Approximately 20% of strains (22/109) were not killed in the hSBA using pooled serum from rL2068 vaccinees, including 11% (10/92) of strains with an MFI of >1,000. The reason why some strains with high levels of fHbp expression are not susceptible to killing in an hSBA warrants additional exploration. For example, information about fHbp sequence variability between susceptible and nonsusceptible strains might help to explain the low observed correlation, in which case, division of strains into those with less than or greater than 90% sequence homology to the vaccine strain’s fHbp amino acid sequence would be illustrative.

McNeil et al. used pooled postvaccination serum from five 18- to 25-year-olds who had received three doses of 20 µg, 60 µg, or 200 µg of rLP2086 (the final formulation contained 120 µg) in a phase 1 study (2), with a pooled median hSBA titer of 153. Postvaccination sera collected 1 month after the third dose were likely used for the evaluations; however, investigation of the persistence of antibody levels over a number of years after immunization would provide additional useful information. Since the kinetic of acute invasive meningococcal disease is very rapid, the benefit of an anamnestic response is questionable, and dependency upon directly available serum antibody levels is therefore likely to be of more importance for long-term protection.

Surface expression levels of fHbp by MenB strains as measured using pooled sera from vaccinees in the MEASURE assay appear to be a relevant factor correlated to bactericidal killing susceptibility, although the authors correctly concluded that population-level coverage conferred through vaccination can be inferred only through assessment of bactericidal activity of sera from individual vaccinees and individual vaccines. Is flow cytometry the optimal fHpb testing approach? Given the likely surface exposure of lipidated fHbp, it is very well possible that chemical determination of fHbp by mass spectrometry in relation to the dry weight of cells equally assesses fHbp expression levels.

Rather than using hSBA to confirm susceptibility to antibody-mediated killing, a complement deposition assay might be useful as an intermediate between MFI and the hSBA. An fHbp enzyme-linked immunosorbent assay (ELISA) possibly employing inhibition with bactericidal antibodies might generate results that correlate with MFI, with complement-binding assays, or directly with *in vitro* bactericidal assays, given that the surface location of fHbp is attached to the outer membrane via a lipid tail. A similar approach has previously been taken for *Borrelia* lipo-OspA-related antibody responses (3, 4). Such a standardized fHbp ELISA would offer a more readily available and reproducible assay for wider use.
